# Supplementary material for: From sequence to enzyme mechanism using multi-label machine learning
Source: BMC Bioinformatics. 2014 May 19;15:150. doi: 10.1186/1471-2105-15-150 (PMC4229970; doi:10.1186/1471-2105-15-150)
Supplement: Additional file 2 — Java code of ml2db. Additional file ml2db_code.tar.gz contains the Java source code to run the multi-label machine learning experiments and save the results to database. The code’s Javadoc is included. [file 1471-2105-15-150-S2.zip › additional file 2/ml2db/ml2db/doc/mulan/classifier/transformation/LabelsetPruning.html]

LabelsetPruning


JavaScript is disabled on your browser.


- Overview
- Package
- Class
- Use
- Tree
- Deprecated
- Index
- Help

- Prev Class
- Next Class

- Frames
- No Frames

- All Classes

- Summary:
- Nested |
- Field |
- Constr |
- Method

- Detail:
- Field |
- Constr |
- Method


mulan.classifier.transformation

## Class LabelsetPruning

- java.lang.Object
- - mulan.classifier.MultiLabelLearnerBase
  - - mulan.classifier.transformation.TransformationBasedMultiLabelLearner
    - - mulan.classifier.transformation.LabelPowerset
      - - mulan.classifier.transformation.LabelsetPruning

- All Implemented Interfaces:
  :   java.io.Serializable, MultiLabelLearner, weka.core.TechnicalInformationHandler

  Direct Known Subclasses:
  :   PPT, PrunedSets

  ---

    

  ```
  public abstract class LabelsetPruning
  extends LabelPowerset
  ```

  Common functionality class for the PPT and PS algorithms

  Version:
  :   2012.02.27

  Author:
  :   Grigorios Tsoumakas

  See Also:
  :   Serialized Form

- - ### Constructor Summary

    Constructors

    Constructor and Description

    `LabelsetPruning(weka.classifiers.Classifier classifier,
    int aP)`

    Constructor that initializes learner with base algorithm and main parameter
  - ### Method Summary

    - ### Methods inherited from class mulan.classifier.transformation.LabelPowerset

      `setConfidenceCalculationMethod, setMakePredictionsBasedOnConfidences, setSeed, setThreshold`
    - ### Methods inherited from class mulan.classifier.transformation.TransformationBasedMultiLabelLearner

      `getBaseClassifier, getTechnicalInformation, globalInfo`
    - ### Methods inherited from class mulan.classifier.MultiLabelLearnerBase

      `build, getDebug, isUpdatable, makeCopy, makePrediction, setDebug`
    - ### Methods inherited from class java.lang.Object

      `equals, getClass, hashCode, notify, notifyAll, toString, wait, wait, wait`

- - ### Constructor Detail


    - #### LabelsetPruning

      ```
      public LabelsetPruning(weka.classifiers.Classifier classifier,
                     int aP)
      ```

      Constructor that initializes learner with base algorithm and main parameter

      Parameters:
      :   `classifier` - base single-label classification algorithm
      :   `aP` - number of instances required for a labelset to be included.


- Overview
- Package
- Class
- Use
- Tree
- Deprecated
- Index
- Help

- Prev Class
- Next Class

- Frames
- No Frames

- All Classes

- Summary:
- Nested |
- Field |
- Constr |
- Method

- Detail:
- Field |
- Constr |
- Method
